# A Case-Control Study of Involvement of Oxidative DNA Damage and Alteration of Antioxidant Defense System in Patients with Basal Cell Carcinoma: Modulation by Tumor Removal

**DOI:** 10.1155/2016/5934024

**Published:** 2016-01-13

**Authors:** Lapatsanant Chaisiriwong, Rungsima Wanitphakdeedecha, Panitta Sitthinamsuwan, Somponnat Sampattavanich, Somruedee Chatsiricharoenkul, Woraphong Manuskiatti, Uraiwan Panich

**Affiliations:** ^1^Department of Pharmacology, Faculty of Medicine Siriraj Hospital, Mahidol University, Bangkok 10700, Thailand; ^2^Department of Dermatology, Faculty of Medicine Siriraj Hospital, Mahidol University, Bangkok 10700, Thailand; ^3^Department of Pathology, Faculty of Medicine Siriraj Hospital, Mahidol University, Bangkok 10700, Thailand

## Abstract

Oxidative damage has been suggested to play a role in the pathogenesis of basal cell carcinoma (BCC). This study illustrated an involvement of oxidative DNA damage and changes in antioxidant defenses in BCC by conducting a case-control study (24 controls and 24 BCC patients) and assessing urinary 7,8-dihydro-8-oxo-2′-deoxyguanosine (8-oxo-dGuo), plasma antioxidant defenses including catalase (CAT), glutathione peroxidase (GPx), NQO1, and total superoxide dismutase (SOD) activities, and glutathione (GSH) levels before surgery and 1 month after surgery. 8-oxo-dGuo expressions as well as protein and mRNA expressions of DNA repair enzyme hOGG1 and antioxidant defenses (CAT, GCLC, GPx, Nrf2, and MnSOD) in nonneoplastic epidermis of control and BCC tissues were also determined. This study observed induction in urinary 8-oxo-dGuo, increased 8-oxo-dGuo expression, and reduced hOGG1 protein and mRNA in BCC tissues, decreased activities of CAT, GPx, and NQO1, but elevated SOD activities and GSH levels in BCC patients and reduction of all antioxidant proteins and genes studied in BCC tissues. Furthermore, decreased plasma antioxidant activities in BCC patients were restored at 1 month after operation compared with preoperative levels. Herein, we concluded that BCC patients were associated with oxidative DNA damage and depletion of antioxidant defenses and surgical removal of BCC correlated with improved redox status.

## 1. Introduction

Basal cell carcinoma (BCC) is the most common nonmelanoma skin cancer (NMSC) worldwide, in particular, in fair-skinned population and its incidence has been rising over the past several years [1–3]. Although BCC can be generally diagnosed and treated by surgical excision, it can be destructive and has a significant impact on patients' quality of life as the tumor removal can cause morbidity including functional disability and disfigurement. Therefore, it is of significance to understand pathogenesis of BCC and identify candidate biomarkers, which may be clinically translated into strategies for its detection and prevention. Photo-damage by chronic exposure to ultraviolet radiation (UVR) has been suggested to play a role in the pathogenesis of BCC, which originates from keratinocytes of the epidermal basal layer [4, 5].

Excessive reactive oxygen species (ROS) generated by UVR have been shown to contribute to malignant transformation of keratinocytes into cancerous cells including BCC probably through oxidative DNA damage, defects in DNA repair, and interference with cellular signaling [6, 7]. The oxidative modification of guanine leads to formation of 8-oxo-7,8-dihydro-2′-deoxyguanosine (8-oxo-dGuo), which finally can pair with adenine and cytosine during DNA replication, resulting in GC-TA mutations found to be associated with development of BCC [8]. 8-oxo-dGuo has also been recognized to be the most abundant and potentially mutagenic if not substantially repaired and has thus been developed as a sensitive and stable biomarker for evaluating the degree of oxidative DNA damage. Case-control studies have previously reported that increased urinary 8-oxo-7,8-dihydroguanine (8-oxo-Gua) and 8-oxo-dGuo levels were detected in patients with metastatic head and neck cancer, breast cancer, and lung cancer [9, 10]. Furthermore, impaired DNA repair capacity was suggested to associate with enhanced susceptibility to cancer and deficiency in DNA repair enzyme, human 8-oxoguanine DNA N-glycosylase 1(hOGG1), a key enzyme responsible for 8-oxo-dGua repair, may also be involved in carcinogenesis [11]. It has been proposed that promotion of antioxidant defenses including catalase (CAT), glutathione peroxidase (GPx), NAD(P)H: quinone oxidoreductase 1 (NQO1), a crucial detoxifying enzyme, and superoxide dismutase (SOD) as well as redox regulation by nuclear factor-erythroid-2-related factor 2 (Nrf2) that can cope with excessive ROS formation in human body may be useful to prevent ROS-mediated neoplastic transformation of various tissues including the skin [12, 13]. A link between increased oxidative DNA damage/decreased antioxidant defense capacity and cancer has been proposed and oxidative/antioxidant defense status has thus been intensively investigated as promising biomarkers in cancer [14–16]. However, relationship between the oxidative DNA damage/antioxidant defense parameters and BCC and whether surgical removal of tumors affects the redox status in BCC patients have not yet been reported. Here, we conducted a case-control study to investigate whether BCC was associated with the oxidative DNA damage, hOGG1 levels, and antioxidant defense status and whether tumor removal affected the redox status of patients with BCC compared to control subjects with nonmalignant skin diseases.

## 2. Methods

### 2.1. Study Population

This case-control study involving 48 Thai subjects (mean age, 66 years; range, 39–87 years, 22 males and 26 females) was approved by the ethics committee of the Siriraj Institutional Review Board (SIRB), Faculty of Medicine Siriraj Hospital, Mahidol University, and written informed consent was obtained by all participants. Case group comprising 24 patients newly diagnosed with BCC (mean age, 67 years; range, 41–87 years, 9 males and 15 females) and control group comprising 24 patients with nonmalignant skin diseases (mean age, 66 years; range, 39–82 years, 13 males and 11 females) were recruited from the outpatient clinic of the Department of Dermatology, Faculty of Medicine, Siriraj Hospital, from 2011 to 2014 and every diagnosis was confirmed by a pathologist. Twenty-four control subjects diagnosed with nonmalignant skin diseases were patients with dermatitis (8) (6 males and 2 females), fibroepithelial polyp (3) (3 males), melanocytic nevus (3) (3 females), normal skin (4) (4 females), and seborrheic keratosis (6) (4 males and 2 females).

### 2.2. Data Collection

Data were collected on demographics, clinical characteristics, and lifestyle as shown in Table 1. Subjects were excluded from the study if they had abnormal clinical characteristics for complete blood count (CBC), blood urea nitrogen (BUN), creatinine (Cr), fasting blood sugar (FBS), cholesterol, high density lipoprotein (HDL), low density lipoprotein (LDL), triglyceride, aspartate transaminase (AST), and alanine transaminase (ALT), had history of chronic diseases such as cancer, metabolic diseases including uncontrolled diabetes mellitus, hypertension, and hyperlipidemia, were under concurrent usage of steroid drugs, had body mass index (BMI) > 30 kg/m^2^, and had history of smoking or drinking alcohol on a regular basis.

All subjects underwent surgical intervention and the collection of blood, urine, and skin tissue samples was done on surgery day prior to operation. At 1 month after surgery, blood samples were collected for evaluation of antioxidant defense parameters and the urinary 8-oxo-dGuo levels of BCC patients were determined at both 1 and 6 months after surgery.

### 2.3. Blood Samples Processing

Blood samples were collected: 2 mL in sodium fluoride tube, 2 mL in EDTA tube, and 6 mL in lithium heparin tube for clinical chemistry assays and 10 mL in EDTA tube for antioxidant assays. Whole blood was processed according to standard protocols and centrifuged at 1,000 ×g for 10 minutes at 4°C, and the supernatants were divided into 750 *μ*L aliquots. Plasma samples were stored at −80°C until testing.

### 2.4. Urine Samples Processing

20 mL of fresh urine samples was collected in urine container tube: 10 mL for clinical chemistry assays and 10 mL for oxidative DNA damage assay. Urine samples were processed according to standard protocols and centrifuged at 3,000 ×g for 10 min at 4°C, and the supernatants were collected and stored at −80°C until testing.

### 2.5. Tissue Samples Processing

The skin tissue samples were obtained from lesions of BCC and nonmalignant skin diseases. All studied skin lesions in case and control patients were located on the sun-exposed areas and the size of all excised tissues was 0.5 cm in diameter. Tissue samples were divided into 2 parts; the first part was fixed in formalin solution and then embedded in paraffin block for immunohistochemistry and the second part was fixed in liquid nitrogen for RT-PCR until testing.

### 2.6. Determination of 8-Oxo-dGuo Levels in Urine by ELISA

Urinary 8-oxo-dGuo level was quantified using a competitive enzyme immunoassay (STA-320, Cell Biolabs, San Diego, CA). Briefly, urine samples (1 : 20 dilution) or 8-oxo-dGuo standards were first added to an 8-oxo-dGuo/BSA conjugate preabsorbed enzyme immunoassay plate. After incubation, an anti-8-oxo-dGuo monoclonal antibody was added, followed by a secondary reaction with a horseradish peroxidase-conjugated antibody. 8-oxo-dGuo levels in the urine samples were then determined by comparison with the 8-oxo-dGuo standard curve. 8-oxo-dGuo levels in the urine of each subject were adjusted by urinary creatinine level and were measured as ng/mg creatinine.

### 2.7. Determination of Antioxidant Defense Status in Plasma

Catalase (CAT) activity was determined following the kit protocol from Cayman Chemical (Ann Arbor, MI). The assay was based on the reaction of methanol and the enzyme in the presence of an optimal concentration of H_2_O_2_. The formaldehyde produced was measured colorimetrically at 540 nm using 4-amino-3-hydrazino-5-mercapto-1,2,4-triazole (Purpald) as the chromogen. CAT activity was expressed in unit/mg protein.

Glutathione peroxidase (GPx) activity assay was performed following manufacturer's instruction (Trevigen, Gaithersburg, MD). GPx activity was coupled to glutathione reductase (GR), which catalyzed NADPH-mediated reduction of GSSG back to GSH as previously described [17]. The rate of NADPH oxidation by H_2_O_2_ was monitored at 340 nm and GPx activity was expressed in unit/mg protein.

NQO1 activity was evaluated spectrophotometrically as previously described [18] using 2,6-dichloroindophenol (DCPIP) as a substrate. The assay was based on the activities for NAD(P)H-dependent reduction of DCPIP at 600 nm and the reaction was specifically inhibited by dicumarol. The NQO1 activity was thus measured as the dicumarol-inhibitable reduction in absorbance at 600 nm and was expressed as nmole DCPIP reduced/min/mg protein.

Assay for measurement of total superoxide dismutase (SOD) activity was modified following the method of Johns et al. [19, 20] and the kit protocol from Cayman Chemical (Ann Arbor, MI). Superoxide anions (O_2_^•−^) were generated by a xanthine/xanthine oxidase (XOD) system and were detected using 3′-{1-[(phenylamino)-carbonyl]-3,4-tetrazolium}-bis(4-methoxy-6-nitro)benzenesulfonic acid (XTT) as the chromogen. The total SOD activity was determined by a decrease in XTT reaction rate at 470 nm as a result of superoxide produced by xanthine/XOD system. The SOD activity was calculated using the following equation: [{(0.953) × ((Std.conc. 0 − Blank)/(Sample − Blank)) − 0.097} × Dilution factor]. Total SOD activity was expressed in unit/mg protein, where one unit of activity was the amount of protein required for a 50% decrease in the rate of XTT reduction.

GSH assay was carried out by glutathione reductase: DTNB enzymatic recycling method following the kit protocol from Sigma-Aldrich (MO, US). Determination of GSH levels involves GSH oxidation by the sulfhydryl reagent DTNB (5,5′-dithio-bis-2-(nitrobenzoic acid)) to produce the yellow TNB (5′-thio-2-nitrobenzoic acid) measured at 412 nm. The glutathione disulfide (GSSG) formed can be recycled to GSH by glutathione reductase in the presence of NADPH. The rate of TNB production is directly proportional to this recycling reaction in turn directly proportional to the concentration of GSH. The GSH level was expressed in nmol/mg protein.

### 2.8. Determination of Protein Content in Plasma by Bradford Assay

Protein concentration was measured using the Bio-Rad Protein Assay Kit (Bio-Rad, Munich, Germany) and bovine serum albumin (BSA) was used as protein standard.

### 2.9. Immunohistochemical (IHC) Determination of Oxidative DNA Damage, Antioxidant Enzyme, and hOGG1 Expression in Skin Tissues

Paraffin-embedded tissues were sectioned (2 *μ*m thickness) and placed on Super-FrostPlus glass slides fixed at 60°C overnight. The sections were deparaffinized in xylene and rehydrated in ethanol series and then incubated in citrate buffer, pH 6 (DaKo). Slides were incubated with 3% hydrogen peroxide and then with 2% BSA. Slides were incubated with primary antibodies against 8-oxo-dGuo (ab48508; Abcam, Cambridge, UK) (1 : 50), hOGG1 (NB100-106, Novus Biologicals, USA) (1 : 100), CAT (ab125688, Abcam, Cambridge, UK) (1 : 200), GCLC (ab53179; Abcam, Cambridge, UK) (1 : 100), GPx (ab108429, Abcam, Cambridge, UK) (1 : 50), and Nrf2 (ab31163, Abcam, Cambridge, UK) (1 : 50). The slides were then incubated with Dako REAL EnVision Detection System, Peroxidase/DAB+, Rabbit/Mouse for 30 min. Peroxidase activity was visualized with 3, 3-diamino-benzidine tetrahydrochloride (DAB) as the substrate and hematoxylin. All tissues were stained with hematoxylin-eosin (H&E) for detection of cell structure.

IHC staining of all samples was evaluated visually and scored by a pathologist twice in different days. If the IHC staining evaluated in duplicate gave different IHC scores, visual interpretation of the IHC staining would be repeated. The semiquantitative analysis of the stained sections was carried out by light-microscopy, employing the immunoreactive score (IRS) according to the study of Kaemmerer et al. [21]. The level of antibody staining was evaluated by IRS and calculated by multiplying the scores of staining intensity by the percentage of positive cells. Based on the IRS, antibody staining pattern was defined as IRS-classification score.

### 2.10. Quantitative Real-Time Reverse Transcription-Polymerase Chain Reaction: Determination of hOGG1, Catalase, GCLC, GCLM, GPx, Nrf2, CuSOD, and MnSOD

Improm-IITM reverse transcriptase (Promega, Madison, USA) was used to synthesize cDNA from total RNA following the manufacturer's protocol. Sequences for PCR primer sets of genes studied were designed using the Primer Express version 3.0 software (Applied Biosystems, USA). Sequences of PCR primer (in 5′→3′ direction) were as follows: hOGG1 (product sizes = 164 bp) sense, TGGAAGAACAGGGCGGGCTA, and antisense, ATGGACATCCACGGGCACAG; CAT (product sizes = 148 bp) sense, CCTTCGACCCAAGCAACATG, and antisense, CGAGCACGGTAGGGACAGTTC; GCLC (product sizes = 160 bp), sense GCTGTCTTGCAGGGAATGTT, and antisense, ACACACCTTCCTTCCCATTG; GCLM (product sizes = 200 bp) sense, TTGGAGTTGCACAGCTGGATT, and antisense, TGGTTTTACCTGTGCCCACTG; GPx (product sizes = 94 bp) sense, ACGATGTTGCCTGGAACTTT, and antisense, TCGATGTCAATGGTCTGGAA; Nrf2 (product sizes = 161 bp) sense, TTCTGTTGCTCAGGTAGCCCCTCA, and antisense, GTTTGGCTTCTGGACTTGG; CuSOD (product sizes = 109 bp) sense, TGCTGGTTTGCGTCGTAGTC, and antisense, ACGCACACGGCCTTCGT; MnSOD (product sizes = 141 bp) sense, TGGCCAAGGGAGATGTTACAG, and antisense, CTTCCAGCAACTCCCCTTTG; GAPDH (product sizes = 150 bp) sense, CCTCCAAAATCAAGTGGGGCGATG, antisense, CGAACATGGGGGCATCAGCAGA. Real-time PCR was performed using FastStart universal SYBR Green Master with ROX (Roche diagnostic, USA) and mRNA expression was quantified by real-time PCR using ABI prism 7300 Real-Time PCR System (Applied Biosystems, USA). Melt curve analysis was performed to verify specificity of the amplified product. mRNA expression was normalized to the expression of GAPDH gene. The mean Ct of each gene in each sample was compared with the mean Ct from GAPDH determinations from the same cDNA sample in order to assess mRNA expression. Ct values were then used to calculate fold change in gene expression.

### 2.11. Statistical Analysis

Descriptive statistics were reported as frequencies and percentage. Categorical variables were analyzed by the chi-square test and performed with SPSS 18.0 software (SPSS, Chicago, IL). Data were expressed as means ± standard deviation (SD). The results were subjected to statistical analysis using Prism 5.0 (GraphPad Software, La Jolla, CA, USA). The Shapiro-Wilk test was employed to test the normal distribution. The statistical significance between nonparametric variables was analyzed by Mann Whitney *U* test or Kruskal-Wallis test and between parametric variables by unpaired Student's *t*-test or one-way analysis of variance (ANOVA) followed by Tukey's* post hoc* test. *P* values less than 0.05 were considered statistically significant. To investigate systematically similarity of biochemical profiles, we performed the principal component analysis using JMP Pro. Data were then plotted using custom MATLAB code.

## 3. Results

### 3.1. Demographic, Lifestyle, and Clinical Characteristics of the Study Subjects

The results shown in Table 1 indicate that the demographic and clinical characteristics are similar.

### 3.2. The Urinary 8-Oxo-dGuo Levels and Antioxidant Defense Status in BCC and Control Subjects

**Figure 1:**
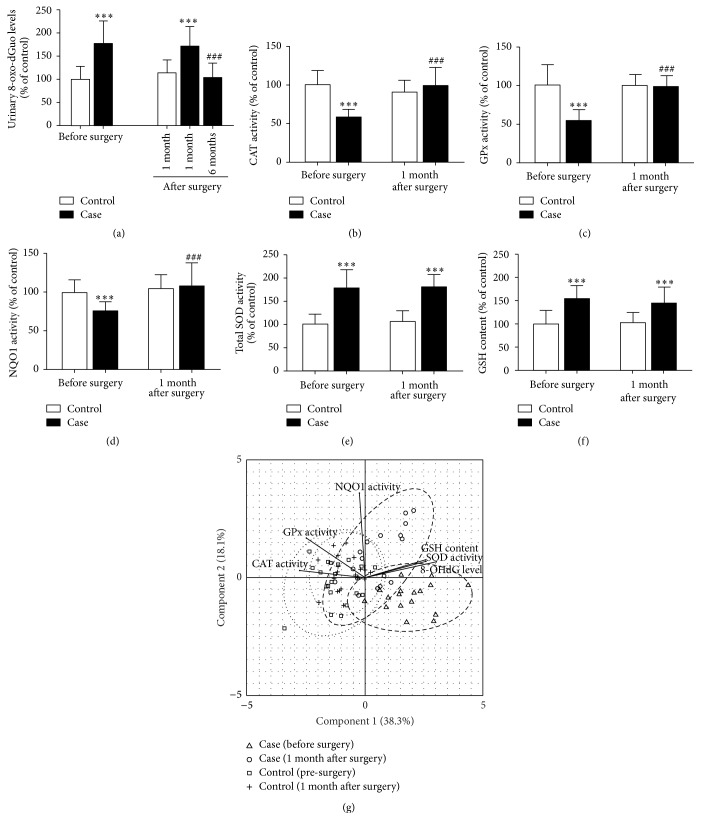
The urinary 8-oxo-dGuo levels (a) and plasma antioxidant defense status [CAT (b), GPx (c), NQO1 (d), and total SOD (e) activities and GSH levels (f)] in control subjects and BCC patients before and after surgery. Values given are mean ± SD. The statistical significance of differences between the control and case was evaluated by one-way ANOVA followed by Tukey's* post hoc* test. ^*∗∗∗*^ *P* < 0.001 compared to preoperative values in control subjects prior to surgery; ^###^ *P* < 0.001 compared to preoperative values in BCC patients prior to surgery. Principal component analysis was performed to systematically investigate similarity of DNA damage and antioxidant defense parameters from different groups of patients (g). Factor analysis is overlaid on top of the patient scores, both before surgery (case: triangle; control: square) and 1 month after surgery (case: circle; control: plus). The dashed and dotted ovals delineate the approximated distribution of each group with 95% confidence intervals.

The urinary 8-oxo-dGuo levels and all antioxidant defense parameters studied in BCC and control subjects were shown in Table 2. Oxidative DNA damage was significantly higher in BCC patients compared to control subjects (Figure 1). After surgery, urinary 8-oxo-dGuo levels of BCC patients were insignificantly altered at 1 month, although they were substantially reduced at 6 months compared to preoperative levels (Figure 1(a)). In addition, while preoperative values of plasma CAT, GPx, and NQO1 activities were observed to be lower in BCC patients compared to control (Figures 1(b)–1(d)), plasma total SOD activities and GSH levels were substantially higher in BCC patients compared to control (Figures 1(e)-1(f)). In comparison between preoperative and postoperative levels of plasma antioxidant defense status in BCC patients, CAT, GPx, and NQO1 activities were increased at 1 month postoperatively, although total SOD activities and GSH levels remained unchanged.

In control subjects, urinary 8-oxo-dGuo levels and all antioxidant defense parameters studied were not significantly different before and after surgery.

To compare systematically the DNA damage and antioxidant defense parameters among different treatment conditions, we utilized the principal component analysis. In the PCA space (Figure 1(g)), data from all patients will be projected into a 2-dimensional plane that can best demonstrate variation among different treatment groups. Specifically, the first and the second components are linearly combined scores from all measurement parameters that can best explain variances observed from all patients, 38.3% and 18.1%, respectively. Based on this calculation, we clearly observed similarity of PCA scores between the two control groups, either before surgery or at 1 month after surgery. On the other hand, patients with BCC clearly exhibit changes of PCA scores after surgery. Interestingly, half of the BCC patients at 1 month after surgery already exhibit PCA profiles that are similar to those of the control groups while the other half still exhibit PCA scores that do not overlap with those from the control groups. This finding implies the potential heterogeneity of antioxidant defense response among patients but shows that antioxidant defense status in BCC patients can be rescued to the level similar to that of the control group patients as early as 1 month after surgery.

### 3.3. IHC Determination of Oxidative DNA Damage, Antioxidant Enzyme, and hOGG1 Expressions in Skin Tissues of BCC and Control Subjects

**Figure 2:**
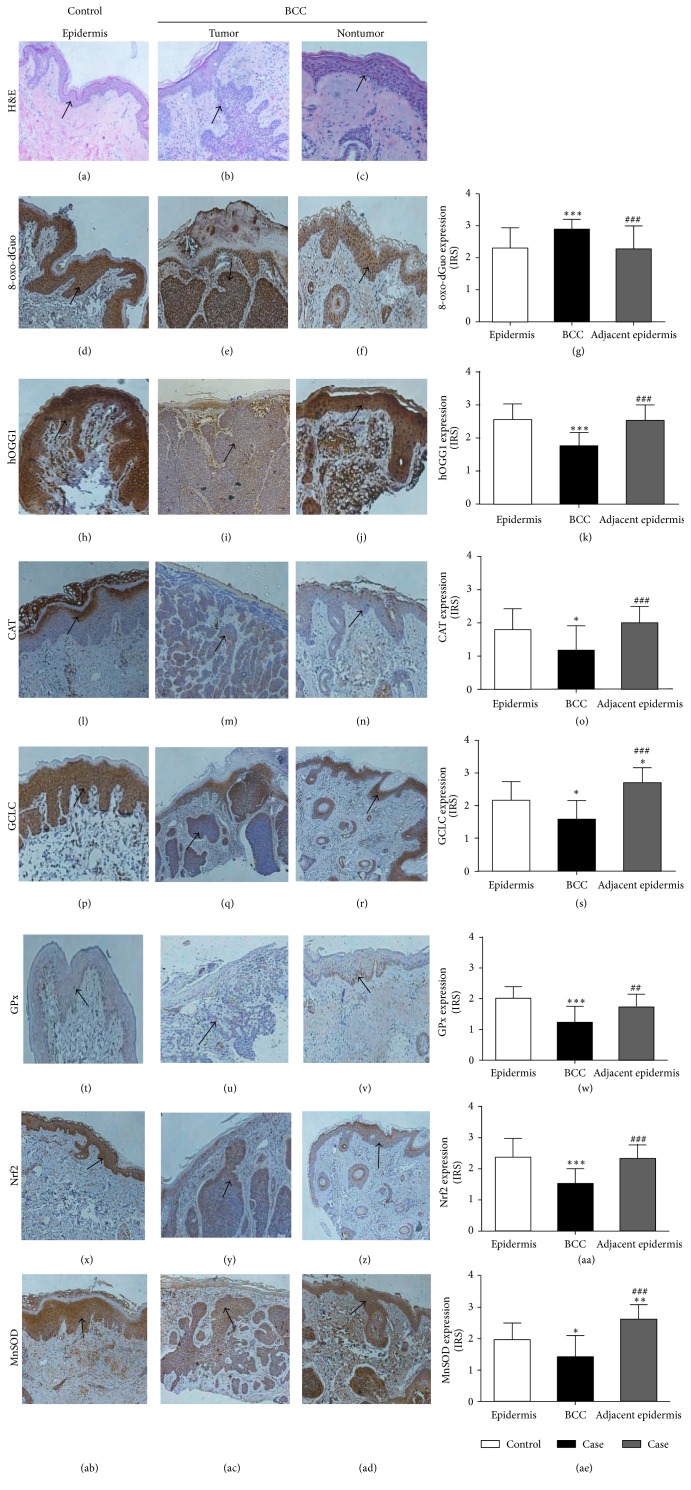
The H&E staining ((a)–(c)) and IHC staining for oxidative DNA damage, 8-oxo-dGuo ((d)–(g)), DNA repair enzyme, hOGG1 ((h)–(k)), and antioxidant proteins, CAT ((l)–(o)), GCLC ((p)–(s)), GPx ((t)–(w)), Nrf2 ((x)–(aa)), and MnSOD ((ab)–(ae)), in control subjects and tumor and nontumor lesions of BCC patients. Values given are mean ± SD. The statistical significance of differences between the control and case and between adjacent epidermis and tumor lesions of BCC patients was evaluated by nonparametric variables with Kruskal-Wallis test followed by Dunnett's* post hoc* test. ^*∗*^ *P* < 0.05, ^*∗∗*^ *P* < 0.01, ^*∗∗∗*^ *P* < 0.001 compared to control; ^##^ *P* < 0.01, ^###^ *P* < 0.001 compared to tumor lesions of BCC patients.

The IHC staining was classified as negative, weak positive, mild positive, and strong positive and the data were presented as average IRS. H&E staining identified structures of skin sections of case and control subjects as shown in Figures 2(a)–2(c).  Table 3 demonstrated that expressions of nuclear 8-oxo-dGuo (Figures 2(d), 2(e), and 2(g)) were higher and hOGG1 proteins (Figures 2(h), 2(i), and 2(k)) in nucleus and cytoplasm were lower in BCC tissues than those in the epidermis of control subjects. Additionally, protein expressions of CAT (Figures 2(l), 2(m), and 2(o)), GCLC (Figures 2(p), 2(q), and 2(s)), GPx (Figures 2(t), 2(u), and 2(w)), Nrf2 (Figures 2(x), 2(y), and 2(aa)), and MnSOD (Figures 2(ab), 2(ac), and 2(ae)) were observed to be lower in BCC tissues compared to the epidermis of control subjects. Furthermore, the intrasubject comparison of BCC and adjacent epidermis demonstrated a higher expression of 8-oxo-dGuo (Figures 2(e), 2(f), and 2(g)) and a lower expression of hOGG1 (Figures 2(i), 2(j), and 2(k)), CAT (Figures 2(m), 2(n), and 2(o)), GCLC (Figures 2(q), 2(r), and 2(s)), GPx (Figures 2(u), 2(v), and 2(w)), Nrf2 (Figures 2(y), 2(z), and 2(aa)), and MnSOD (Figures 2(ac), 2(ad), and 2(ae)) in BCC lesions compared to adjacent normal skin. In consistent with our studies for plasma antioxidant defense status showing higher total SOD activities and GSH levels in BCC patients compared with control subjects, protein expressions of GCLC and MnSOD were higher in nonneoplastic tissues of BCC patients compared to both BCC tissues and the epidermis of control subjects.

In comparison of noncancerous skin lesions and normal skin of control subjects, there were no significant differences in expressions of all parameters studied (data not shown).

### 3.4. mRNA Expression of hOGG1 and Antioxidant Defense System in Skin Tissues of BCC and Control Subjects

**Figure 3:**
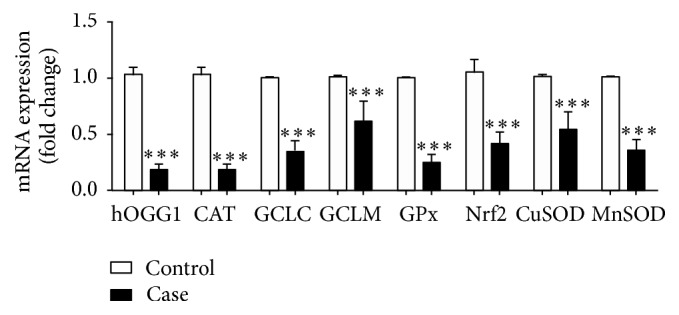
DNA repair and antioxidant gene expression in nonmalignant skin tissues of control subjects and BCC tissues. Gene expression was evaluated by real-time PCR with the 2^−ΔΔCt^ method. The data are presented as the fold change in gene expression normalized to GAPDH. Values given are mean ± SD. The statistical significance of differences between the control and case was evaluated by nonparametric variables with Mann Whitney *U* test. ^*∗∗∗*^ *P* < 0.001 compared to control.

In agreement with protein expression data, Figure 3 demonstrated that mRNA expressions of hOGG1 (0.85 ± 0.12-fold decrease, *P* < 0.001), CAT (0.88 ± 0.03-fold decrease, *P* < 0.001), GCLC (0.66 ± 0.09-fold decrease, *P* < 0.001), GCLM (0.40 ± 0.17-fold decrease, *P* < 0.001), GPx (0.76 ± 0.07-fold decrease, *P* < 0.001), Nrf2 (0.63 ± 0.02-fold decrease, *P* < 0.001), CuSOD (0.47 ± 0.13-fold decrease, *P* < 0.001), and MnSOD (0.65 ± 0.09-fold decrease, *P* < 0.001) were substantially lower in BCC tissues than in skin tissues of control subjects.

## 4. Discussion

An involvement of oxidative damage in the pathogenesis of BCC has been widely discussed since several studies have shown possible mechanisms through which excessive ROS generation and antioxidant defense impairment may play a role in malignant transformation to NMSC or keratinocytic cancer including BCC [6, 22]. Potential mechanisms of carcinogenesis may involve oxidative DNA damage accountable for genomic mutations because attacks on DNA by ROS result in considerable DNA lesions including strand breaks and DNA base oxidation products, in particular 8-oxo-dGuo, considered the most potentially mutagenic [23]. hOGG1 is highly specific for the removal and repair of 8-oxo-dGuo from oxidatively damaged DNA. Increased 8-oxo-dGuo formation and/or loss of hOGG1's expression and function were reported to play a role in the development and progression of skin cancers including BCC [24–26]. In this study, we observed elevation of preoperative 8-oxo-dGuo levels in urine and its expression in BCC tissues in comparison with epidermis of control subjects. At 1 month after operation, there were no significant changes in urinary 8-oxo-dGuo levels in BCC patients compared with preoperative levels, although the postoperative 8-oxo-dGuo levels in BCC patients were substantially reduced at 6 months. It is possible that greater urinary 8-oxo-dGuo in BCC patients than in control subjects observed in our study could be attributed to oxidative DNA damage in neoplastic cells of BCC tissues. Changes in system redox status in patients subjected to surgical removal of primary cutaneous tumor were also reported. Gadjeva et al. observed higher malondialdehyde (MDA) levels, lipid peroxidation products as an oxidative stress marker, in plasma of patients with melanoma compared with control subjects and reduction of the MDA levels at 20 days after surgery compared with preoperative values [27].

In addition, urinary 8-oxo-dGuo levels in control subjects in our study were observed to be higher than those of healthy subjects in previous studies employing ELISA [28, 29], although the levels found in this study were comparable to those reported by Ksiazek et al. [30]. The urinary 8-oxo-dGuo levels were usually higher and variable when detected by ELISA compared with chromatographic methods. Thus, further studies using chromatographic techniques including chromatographic techniques with either mass spectrometric or electrochemical detection having higher sensitivity and specificity for determination of urinary 8-oxo-dGuo levels need to be done with a larger sample size in order to confirm an association between oxidative DNA damage and BCC.

We also observed decreased protein and mRNA expressions of hOGG1 in BCC tissues in comparison with epidermis of control subjects. Furthermore, the intrasubject comparison of nonneoplastic epidermis adjacent to BCC and lesional BCC skin demonstrated a higher expression of 8-oxo-dGuo and a lower expression of hOGG1 in BCC tissues compared with the adjacent epidermis. Our results are consistent with those from previous case-control studies suggesting a contribution of oxidative DNA damage and impaired hOGG1 to the development of various cancers including breast, pancreatic, gastric, and lung cancers [9, 31–33]. Hence, augmented levels of 8-oxo-dGuo could be related to a defect in hOGG1 expression in BCC patients that may be deficient in the repair of 8-oxo-dGuo, although the mechanism by which hOGG1 impairment contributes to the development of BCC needs further investigation.

A disturbance in redox homeostasis probably contributed to development of multiple tumors including NMSC which can be attributed to not only increased oxidative DNA damage but also impaired antioxidant defense capacity [6, 34]. Major antioxidant defenses in the human body include GSH and enzymatic antioxidants including GPx and CAT, which neutralize H_2_O_2_, NQO1, which catalyzes detoxification of various electrophilic toxicants and oxidants and SOD, which dismutates the superoxide radical (O_2_^•−^) to H_2_O_2_. Thus, pre- and postoperative plasma levels of GSH levels and antioxidant enzyme (CAT, GPx, NQO1, and SOD) activities as well as protein and mRNA expressions of related antioxidant enzymes (GCLC, CAT, GPx, and MnSOD) and Nrf2, a well-known transcription factor that regulates expression of detoxifying enzyme genes, in skin lesions were determined in BCC patients compared with control subjects. Our results indicated that while decreases in CAT, GPx, and NQO1 activities in BCC patients and in protein expressions of CAT, GCLC, GPx, Nrf2, and MnSOD in BCC tissues compared to epidermis of control subjects were observed, plasma SOD activities and GSH levels were higher in BCC patients than in control subjects. In correlation with IHC findings showing diminished expressions of all antioxidant proteins in BCC tissues, mRNA levels of the corresponding genes including CAT, GCLC, GCLM, NQO1, Nrf2, CuSOD, and MnSOD were also reduced in BCC tissues compared to epidermis of control subjects.

While CAT, GPx, and NQO1 activities markedly declined in BCC patients compared to control subjects, elevation of SOD activities and GSH levels was observed in association with upregulated protein expressions of GCLC, a rate-limiting enzyme in GSH synthesis, and MnSOD in the adjacent nonneoplastic tissues of BCC patients. In agreement with previous studies, lower activities of GPx and CAT as well as higher activities of SOD were observed in patients with oesophageal, gastric, and colorectal cancers compared with control subjects [35, 36] and low NQO1 activity was linked to increased risk of acute leukemia [37]. NQO1 polymorphism, which can cause reduction of its activity, was reported to affect susceptibility to lung, bladder, and colorectal cancers that could either increase or decrease cancer risks associated with ethnicity and exposure to carcinogens [38]. In addition, this study demonstrated decreased Nrf2 expression in BCC tissues compared to both adjacent nonneoplastic tissues of BCC patient and noncancerous tissues of control subjects which was in agreement with previous reports showing downregulation of Nrf2 and its target genes including NQO1 was associated with malignant transformation and upregulation of antioxidant mRNA including CAT, GCLC, GCLM, GPx, and NQO1 genes by Nrf2 overexpression was able to delay tumor growth [39]. Nevertheless, the role of Nrf2 in cancer is complex as it can play a dual role in both cancer prevention and promotion depending on cellular environment [40]. In addition, differential expression of antioxidant proteins was found in several cancers. Reduction of CAT and GPx protein expressions was demonstrated in neoplastic tissues of patients with esophageal, colorectal, and lung cancers, although both downregulation and overexpression of MnSOD protein in lung and esophageal cancers, respectively, were reported [41–43].

Enhancement of plasma SOD activities and GSH levels in correlation to increased protein expressions of MnSOD and GCLC in the adjacent nonneoplastic tissues of BCC patients may be due to an adaptive response of normal skin cells to persistent elevation of oxidative stress and damage in cancer patients. It has been suggested that upregulation of antioxidant defenses including GSH and MnSOD may serve as the defense mechanisms for cell survival against stress and inflammatory insults, which can take place during cancer initiation and progression [44]. Accumulation of ROS and oxidative damage during malignant transformation was found to be accountable for transcriptional downregulation of antioxidant defense via Nrf2 signaling [39]. Moreover, previous reports suggested that the presence of a tumor could have the systemic impact on distant tissues by generating oxidative stress and releasing inflammatory mediators including cytokines, which in turn can promote oxidative damage [45, 46]. Thus, increased oxidative damage and changes in antioxidant defense system may associate with initiation of carcinogenesis and/or occur as a consequence of alterations in cellular homeostasis and antioxidative metabolism or of inflammation in tumor growth, progression, and microenvironment. Case-control studies of genetic polymorphisms in DNA repair and antioxidant enzymes as well as changes in inflammatory parameters in association with BCC risk with a large number of subjects should also be done in future studies.

This study showed that patients with BCC, a locally invasive malignant skin cancer, could exhibit systemic disturbance in redox status. Previous* in vivo* and clinical studies have reported the impact of skin damage on systemic oxidative parameter. Acute exposure of mice skin to UVA and UVB irradiation was shown to affect markers of oxidative damage and antioxidant defenses in plasma and nonskin tissues including erythrocytes and liver [47]. Moreover, lower CAT activity and higher SOD activity and MDA levels were observed in plasma of patients with acne vulgaris [48] and lower GSH levels and greater MDA levels in plasma of cutaneous melanoma patients compared with control subjects [49]. Furthermore, our findings suggested that surgical intervention could influence oxidative DNA damage and antioxidant defense status only in case patients with BCC but not control subjects with nonmalignant skin diseases because tumor removal was observed to be related to improvement of redox status by reducing 8-oxo-dGuo levels at 6 months postoperatively and enhancing plasma antioxidant defenses including CAT, GPx, and NQO1 activities at 1 month postoperatively.

## 5. Conclusions

Patients with BCC may be under oxidative stress associated with induction of oxidative DNA damage, defects in DNA repair hOGG1 at protein and mRNA levels, and reduction of plasma CAT, GPx, and NQO1 activities and of all antioxidant proteins and genes studied in the BCC tissues. Surgical removal of BCC tissues correlated with improved redox status. An elevation of plasma total SOD activities and GSH levels as well as protein expressions of MnSOD and GCLC in nonneoplastic tissues of BCC patients may indicate an adaptive response to oxidative stress. Whether oxidative DNA damage and antioxidant defense parameters can serve as biomarkers of oxidative stress to predict development and progression of BCC needs further studies.

## Figures and Tables

**Table 1:** Demographics and clinical characteristics of controls subjects and BCC patients.

Characteristics	Control (*n* = 24)	Cases (*n* = 24)	*P* value
*Demographic*			
Age (years)	64.71 ± 10.58	66.82 ± 11.59	0.714^b^
Gender, *n* (%)			
Male	13 (54.20)	9 (37.5)	0.247^a^
Female	11 (45.8)	15 (62.5)	
BMI (kg/m^2^)	23.41 ± 2.38	22.98 ± 3.31	0.217^b^
*Clinical characteristics*			
Glucose (mg/dL)	95.82 ± 12.24	101.65 ± 14.10	0.317^c^
BUN (mg/dL)	13.44 ± 3.82	14.32 ± 5.09	0.891^b^
Creatinine (mg/dL)	1.04 ± 0.23	1.05 ± 0.25	0.949^c^
Cholesterol (mg/dL)	187.97 ± 36.44	201.76 ± 34.21	0.402^c^
Triglyceride (mg/dL)	129.64 ± 62.23	109.76 ± 43.34	0.156^b^
HDL-Chol (mg/dL)	57.88 ± 18.92	63.59 ± 17.27	0.402^b^
LDL-Cal (mg/dL)	101.85 ± 31.34	116.22 ± 31.06	0.110^b^
AST (U/L)	27.24 ± 12.01	26.82 ± 13.50	0.359^b^
ALT (U/L)	20.67 ± 9.32	21.29 ± 12.68	0.593^b^
eGFR (mL/min/1.73 m^2^)	64.83 ± 19.44	65.24 ± 18.94	0.849^c^
CBC			
Hemoglobin (g/dL)	13.21 ± 1.76	12.84 ± 1.25	0.200^b^
RBC count (×10^6^/*µ*L)	4.60 ± 0.62	4.49 ± 0.48	0.404^c^
WBC count (×10^3^/*µ*L)	6.91 ± 1.65	7.63 ± 1.48	0.195^b^
*Lifestyle *			
Smoking, *n* (%)			
Nonsmoker	18 (75.0)	19 (79.2)	0.598^a^
Ex-smoker	5 (20.8)	5 (20.8)	
Current smoker	1 (4.2)	0 (0)	
Drinking alcohol, *n* (%)			
Nondrinker	19 (79.2)	19 (79.2)	0.574^a^
Ex-drinker	4 (16.7)	5 (20.8)	
Occupation type, *n* (%)			
Indoor	22 (91.7)	21 (87.5)	0.637^a^
Outdoor	2 (8.3)	3 (12.5)	
Vegetarian, *n* (%)			
Yes	1 (4.2)	0 (0.0)	0.312^a^
No	23 (95.8)	24 (100.0)	
Chemical, *n* (%)			
Yes	0 (0.0)	1 (4.2)	0.312^a^
No	24 (100.0)	23 (95.8)	

BMI = body mass index, BUN = blood urea nitrogen, HDL-Chol = high density lipoprotein-cholesterol, LDL-Cal = calculated low density lipoprotein cholesterol, AST = aspartate aminotransferase, ALT = alanine aminotransferase, eGFR = estimated glomerular filtration rate, CBC = complete blood count, RBC = red blood cell, and WBC = white blood cell. Data are presented as the mean ± SD. The statistical significance of differences in categorical variables data was evaluated by chi-square test (a); nonparametric variables were analyzed by the Mann-Whitney *U* test (b) and parametric variables by unpaired Student's *t*-test (c).

**Table 2:** Comparison of urinary oxidative DNA damage levels and plasma antioxidant defense status between control subjects and BCC patients.

Parameters	Controls (*n* = 24)		Cases (*n* = 24)		
	Before surgery	1 month after surgery	Before surgery	1 month after surgery	6 months after surgery
8-oxo-dGuo (ng/mg creatinine)	61.92 ± 17.35	71.51 ± 16.68	110.08 ± 30.09^*∗∗∗*^	106.65 ± 26.17^*∗∗∗*^	64.44 ± 19.02^###^
CAT (unit/mg protein)	4.38 ± 0.80	3.96 ± 0.68	2.55 ± 0.43^*∗∗∗*^	4.34 ± 1.13^###^	
GPx (unit/mg protein)	0.77 ± 0.20	0.77 ± 0.21	0.42 ± 0.13^*∗∗∗*^	0.75 ± 0.11^###^	
NQO1 (*µ*mole DCPIP reduced/min/mg protein)	928.25 ± 203.20	977.87 ± 184.54	708.53 ± 112.66^*∗∗∗*^	1010.30 ± 281.00^###^	
Total SOD (unit/mg protein)	0.02 ± 0.01	0.02 ± 0.00	0.04 ± 0.01^*∗∗∗*^	0.04 ± 0.01^*∗∗∗*^	
GSH (*µ*mol/mg protein)	152.19 ± 44.88	156.92 ± 40.41	235.76 ± 42.75^*∗∗∗*^	220.93 ± 52.44^*∗∗∗*^	

Results were expressed as mean ± standard deviation (SD).

^*∗∗∗*^ *P* < 0.001 compared to control, before surgery.

^###^ *P* < 0.001 compared to case, before surgery.

**Table 3:** Expressions of oxidative DNA damage, DNA repair enzyme, and antioxidant proteins in skin tissues of control subjects and BCC patients by IRS.

Parameters	Controls (*n* = 24)	Cases (*n* = 24)	
	Epidermis	BCC	Adjacent epidermis
8-oxo-dGuo	2.30 ± 0.67	2.88 ± 0.30^*∗∗∗*^	2.27 ± 0.72^###^
hOGG1	2.56 ± 0.47	1.77 ± 0.40^*∗∗∗*^	2.53 ± 0.47^###^
CAT	1.79 ± 0.63	1.18 ± 0.74^*∗*^	2.00 ± 0.48^###^
GCLC	2.17 ± 0.57	1.59 ± 0.56^*∗*^	2.70 ± 0.45^*∗*,###^
GPx	2.02 ± 0.37	1.24 ± 0.51^*∗∗∗*^	1.73 ± 0.41^##^
Nrf2	2.40 ± 0.57	1.53 ± 0.47^*∗∗∗*^	2.33 ± 0.44^###^
MnSOD	1.95 ± 0.53	1.41 ± 0.65^*∗*^	2.60 ± 0.46^*∗∗*,###^

Results were expressed as mean ± standard deviation (SD).

^*∗*^ *P* < 0.05, ^*∗∗*^ *P* < 0.01, ^*∗∗∗*^ *P* < 0.001 compared to epidermis of control.

^##^ *P* < 0.01, ^###^ *P* < 0.001 compared to BCC lesion.

## Abbreviations

BCC: Basal cell carcinoma
CAT: Catalase
GCLC: Glutamate-cysteine ligase, catalytic subunit
GCLM: Glutamate cysteine ligase, modifier subunit
GSH: Glutathione
GSSG: Glutathione disulfide
GPx: Glutathione peroxidase
H&E: Hematoxylin-eosin
8-oxo-dGuo: 8-oxo-7,8-dihydro-2′-deoxyguanosine
hOGG1: Human 8-oxoguanine DNA N-glycosylase 1
MDA: Malondialdehyde
NMSC: Nonmelanoma skin cancers
NQO1: NAD(P)H dehydrogenase [quinone] 1
Nrf2: Nuclear factor (erythroid-derived 2)-like 2
ROS: Reactive oxygen species
CuSOD: Cupper superoxide dismutase
MnSOD: Manganese superoxide dismutase
UVR: Ultraviolet radiation.

## References

[1] Christenson L. J., Borrowman T. A., Vachon C. M. (2005). Incidence of basal cell and squamous cell carcinomas in a population younger than 40 years. *The Journal of the American Medical Association*.

[2] Flohil S. C., Seubring I., van Rossum M. M., Coebergh J.-W. W., De Vries E., Nijsten T. (2013). Trends in basal cell carcinoma incidence rates: a 37-year dutch observational study. *The Journal of Investigative Dermatology*.

[3] Lomas A., Leonardi-Bee J., Bath-Hextall F. (2012). A systematic review of worldwide incidence of nonmelanoma skin cancer. *The British Journal of Dermatology*.

[4] Iannacone M. R., Wang W., Stockwell H. G. (2012). Patterns and timing of sunlight exposure and risk of basal cell and squamous cell carcinomas of the skin—a case-control study. *BMC Cancer*.

[5] Xiang F., Lucas R., Hales S., Neale R. (2014). Incidence of nonmelanoma skin cancer in relation to ambient UV radiation in white populations, 1978–2012: empirical relationships. *JAMA Dermatology*.

[6] Sander C. S., Hamm F., Elsner P., Thiele J. J. (2003). Oxidative stress in malignant melanoma and non-melanoma skin cancer. *The British Journal of Dermatology*.

[7] Tilli C. M. L. J., Van Steensel M. A. M., Krekels G. A. M., Neumann H. A. M., Ramaekers F. C. S. (2005). Molecular aetiology and pathogenesis of basal cell carcinoma. *The British Journal of Dermatology*.

[8] Kunisada M., Yogianti F., Sakumi K., Ono R., Nakabeppu Y., Nishigori C. (2011). Increased expression of versican in the inflammatory response to UVB- and reactive oxygen species-induced skin tumorigenesis. *The American Journal of Pathology*.

[9] Rozalski R., Gackowski D., Roszkowski K., Foksinski M., Olinski R. (2002). The level of 8-hydroxyguanine, a possible repair product of oxidative DNA damage, is higher in urine of cancer patients than in control subjects. *Cancer Epidemiology Biomarkers & Prevention*.

[10] Kuo H. W., Chou S. Y., Hu T. W., Wu F. Y., Chen D. J. (2007). Urinary 8-hydroxy-2′-deoxyguanosine (8-OHdG) and genetic polymorphisms in breast cancer patients. *Mutation Research*.

[11] Shinmura K., Yokota J. (2001). The OGG1 gene encodes a repair enzyme for oxidatively damaged DNA and is involved in human carcinogenesis. *Antioxidants & Redox Signaling*.

[12] Acharya A., Das I., Chandhok D., Saha T. (2010). Redox regulation in cancer: a double-edged sword with therapeutic potential. *Oxidative Medicine and Cellular Longevity*.

[13] Bickers D. R., Athar M. (2006). Oxidative stress in the pathogenesis of skin disease. *Journal of Investigative Dermatology*.

[14] Knasmüller S., Nersesyan A., Mišík M. (2008). Use of conventional and -omics based methods for health claims of dietary antioxidants: a critical overview. *British Journal of Nutrition*.

[15] Koedrith P., Seo Y. R. (2011). Advances in carcinogenic metal toxicity and potential molecular markers. *International Journal of Molecular Sciences*.

[16] Loft S., Danielsen P., Løhr M. (2012). Urinary excretion of 8-oxo-7,8-dihydroguanine as biomarker of oxidative damage to DNA. *Archives of Biochemistry and Biophysics*.

[17] Pluemsamran T., Onkoksoong T., Panich U. (2012). Caffeic acid and ferulic acid inhibit UVA-induced matrix metalloproteinase-1 through regulation of antioxidant defense system in keratinocyte HaCaT cells. *Photochemistry and Photobiology*.

[18] Siegel D., Kepa J. K., Ross D. (2007). UNIT 4.22 Biochemical and genetic analysis of NAD(P)H:quinone oxidoreductase 1 (NQO1). *Current Protocols in Toxicology*.

[19] Johns E. J., O'Shaughnessy B., O'Neill S., Lane B., Healy V. (2010). Impact of elevated dietary sodium intake on NAD(P)H oxidase and SOD in the cortex and medulla of the rat kidney. *The American Journal of Physiology—Regulatory Integrative and Comparative Physiology*.

[20] Monk L. S., Fagerstedt K. V., Crawford R. M. (1987). Superoxide dismutase as an anaerobic polypeptide: a key factor in recovery from oxygen deprivation in iris pseudacorus?. *Plant Physiology*.

[21] Kaemmerer D., Peter L., Lupp A. (2012). Comparing of IRS and Her2 as immunohistochemical scoring schemes in gastroenteropancreatic neuroendocrine tumors. *International Journal of Clinical and Experimental Pathology*.

[22] Rizzato C., Canzian F., Rudnai P. (2011). Interaction between functional polymorphic variants in cytokine genes, established risk factors and susceptibility to basal cell carcinoma of skin. *Carcinogenesis*.

[23] Kryston T. B., Georgiev A. B., Pissis P., Georgakilas A. G. (2011). Role of oxidative stress and DNA damage in human carcinogenesis. *Mutation Research/Fundamental and Molecular Mechanisms of Mutagenesis*.

[24] Huang X. X., Scolyer R. A., Abubakar A., Halliday G. M. (2012). Human 8-oxoguanine-DNA glycosylase-1 is downregulated in human basal cell carcinoma. *Molecular Genetics and Metabolism*.

[25] Kunisada M., Sakumi K., Tominaga Y. (2005). 8-Oxoguanine formation induced by chronic UVB exposure makes Ogg1 knockout mice susceptible to skin carcinogenesis. *Cancer Research*.

[26] Murtas D., Piras F., Minerba L. (2010). Nuclear 8-hydroxy-2′-deoxyguanosine as survival biomarker in patients with cutaneous melanoma. *Oncology Reports*.

[27] Gadjeva V., Dimov A., Georgieva N. (2008). Influence of therapy on the antioxidant status in patients with melanoma. *Journal of Clinical Pharmacy and Therapeutics*.

[28] Muzembo B. A., Nagano Y., Eitoku M. (2014). 8-Oxoguanine formation induced by chronic UVB exposure makes Ogg1 knockout mice susceptible to skin carcinogenesis. *Environmental Health and Preventive Medicine*.

[29] Chung W. (2015). The cost of liver disease in Korea: methodology, data, and evidence. *Clinical and Molecular Hepatology*.

[30] Ksiazek K., Piatek K., Witowski J. (2008). Impaired response to oxidative stress in senescent cells may lead to accumulation of DNA damage in mesothelial cells from aged donors. *Biochemical and Biophysical Research Communications*.

[31] Chang C.-H., Hsiao C.-F., Chang G.-C. (2009). Interactive effect of cigarette smoking with human 8-oxoguanine DNA *N*-glycosylase 1 (*hOGG1*) polymorphisms on the risk of lung cancer: a case-control study in Taiwan. *American Journal of Epidemiology*.

[32] Hocevar B. A., Kamendulis L. M., Pu X. (2014). Contribution of environment and genetics to pancreatic cancer susceptibility. *PLoS ONE*.

[33] Ma Y., Zhang L., Rong S. (2013). Relation between gastric cancer and protein oxidation, DNA damage, and lipid peroxidation. *Oxidative Medicine and Cellular Longevity*.

[34] Trachootham D., Lu W., Ogasawara M. A., Valle N. R.-D., Huang P. (2008). Redox regulation of cell survival. *Antioxidants and Redox Signaling*.

[35] Dursun H., Bilici M., Uyanik A., Okcu N., Akyüz M. (2006). Antioxidant enzyme activities and lipid peroxidation levels in erythrocytes of patients with oesophageal and gastric cancer. *Journal of International Medical Research*.

[36] Maffei F., Angeloni C., Malaguti M. (2011). Plasma antioxidant enzymes and clastogenic factors as possible biomarkers of colorectal cancer risk. *Mutation Research/Fundamental and Molecular Mechanisms of Mutagenesis*.

[37] Smith M. T., Wang Y., Kane E. (2001). Low NAD(P)H:quinone oxidoreductase 1 activity is associated with increased risk of acute leukemia in adults. *Blood*.

[38] Chao C., Zhang Z.-F., Berthiller J., Boffetta P., Hashibe M. (2006). NAD(P)H:quinone oxidoreductase 1 (*NQO1*) Pro187Ser polymorphism and the risk of lung, bladder, and colorectal cancers: a meta-analysis. *Cancer Epidemiology Biomarkers &d Prevention*.

[39] Funes J. M., Henderson S., Kaufman R. (2014). Oncogenic transformation of mesenchymal stem cells decreases Nrf2 expression favoring in vivo tumor growth and poorer survival. *Molecular Cancer*.

[40] Moon E. J., Giaccia A. (2015). Dual roles of NRF2 in tumor prevention and progression: possible implications in cancer treatment. *Free Radical Biology and Medicine*.

[41] Ho J. C.-M., Zheng S., Comhair S. A. A., Farver C., Erzurum S. C. (2001). Differential expression of manganese superoxide dismutase and catalase in lung cancer. *Cancer Research*.

[42] Murawaki Y., Tsuchiya H., Kanbe T. (2008). Aberrant expression of selenoproteins in the progression of colorectal cancer. *Cancer Letters*.

[43] Zhang J., Wang K., Zhang J., Liu S. S., Dai L., Zhang J.-Y. (2011). Using proteomic approach to identify tumor-associated proteins as biomarkers in human esophageal squamous cell carcinoma. *Journal of Proteome Research*.

[44] Reuter S., Gupta S. C., Chaturvedi M. M., Aggarwal B. B. (2010). Oxidative stress, inflammation, and cancer: how are they linked?. *Free Radical Biology and Medicine*.

[45] Georgakilas A. G., Redon C. E., Ferguson N. F. (2014). Systemic DNA damage accumulation under in vivo tumor growth can be inhibited by the antioxidant Tempol. *Cancer Letters*.

[46] Landskron G., De la Fuente M., Thuwajit P., Thuwajit C., Hermoso M. A. (2014). Chronic inflammation and cytokines in the tumor microenvironment. *Journal of Immunology Research*.

[47] Svobodová A. R., Galandáková A., Šianská J., Doležal D., Ulrichová J., Vostálováa J. (2011). Acute exposure to solar simulated ultraviolet radiation affects oxidative stress-related biomarkers in skin, liver and blood of hairless mice. *Biological & Pharmaceutical Bulletin*.

[48] Arican O., Kurutas E. B., Sasmaz S. (2005). Oxidative stress in patients with acne vulgaris. *Mediators of Inflammation*.

[49] Bernardes S. S., de Souza-Neto F. P., Ramalho L. N. (2015). Systemic oxidative profile after tumor removal and the tumor microenvironment in melanoma patients. *Cancer Letters*.

